# Endocannabinoid-Mediated Neuromodulation in the Olfactory Bulb: Functional and Therapeutic Significance

**DOI:** 10.3390/ijms21082850

**Published:** 2020-04-19

**Authors:** Naina Bhatia-Dey, Thomas Heinbockel

**Affiliations:** Department of Anatomy, Howard University College of Medicine, Washington, DC 20059, USA; naina.bhatiadey@howard.edu

**Keywords:** Alzheimer’s Disease, endocannabinoids, neurodegeneration, neuromodulation, neural dysfunction, odor, olfactory bulb, olfactory system, synaptic plasticity

## Abstract

Endocannabinoid synthesis in the human body is naturally occurring and on-demand. It occurs in response to physiological and environmental stimuli, such as stress, anxiety, hunger, other factors negatively disrupting homeostasis, as well as the therapeutic use of the phytocannabinoid cannabidiol and recreational use of exogenous cannabis, which can lead to cannabis use disorder. Together with their specific receptors CB1R and CB2R, endocannabinoids are major components of endocannabinoid-mediated neuromodulation in a rapid and sustained manner. Extensive research on endocannabinoid function and expression includes studies in limbic system structures such as the hippocampus and amygdala. The wide distribution of endocannabinoids, their on-demand synthesis at widely different sites, their co-existence in specific regions of the body, their quantitative differences in tissue type, and different pathological conditions indicate their diverse biological functions that utilize specific and overlapping pathways in multiple organ systems. Here, we review emerging evidence of these pathways with a special emphasis on the role of endocannabinoids in decelerating neurodegenerative pathology through neural networks initiated by cells in the main olfactory bulb.

## 1. Introduction

The endocannabinoid system is a unique system of neuromodulation that has been characterized mainly in the last thirty years starting with the identification of its main and associated receptor components, ligands, agonists, antagonists, participating in synthesis and degradation, cofactors, transporter proteins, activating and inhibitory cytoskeletal components, transcription factors and their modifiers [1,2,3]. Both endogenous and exogenous ligands of the endocannabinoid system affect standard physiological processes such as pain, inflammation, nausea, and feeding behavior together with psychoactive functions such as memory, emotion, cognition, and reward [2,3]. In general, endocannabinoids function as retrograde messengers that mediate short-term synaptic plasticity through two distinct mechanisms: depolarization-induced suppression of inhibition (DSI) and depolarization-induced suppression of excitation (DSE). DSI involves a reduction of gamma-aminobutyric acid (GABA) neurotransmitter release from presynaptic neurons resulting in the suppression of inhibition in postsynaptic neurons [4]. DSI has been demonstrated in several brain regions such as the hippocampus, amygdala, and the main olfactory bulb. In the main olfactory bulb, DSI allows olfactory bulb output neurons to be transiently relieved from inhibition, potentially to facilitate the detection of weak odor signals [5,6]. In contrast, DSE leads to reduction of glutamate release, thereby suppressing glutamate-mediated excitation at neural synapses [7]. Both signaling pathways indicate the participation of the endocannabinoid system in a site-specific manner affecting specific neurotransmitter release in each case. Based on site-specific on-demand synthesis in many tissues and the involvement of multiple cell types where retrograde messenger activity affects synaptic plasticity, there is a surge of research activity to identify endocannabinoid functions in neurodegenerative diseases in a bidirectional approach: first, in disrupting the progression of symptoms of neurodegenerative pathology and second, in applying therapeutic intervention/s to modify erratic behavioral patterns that may emerge as consequence of progressing neurodegenerative pathology. There is extensive experimental evidence regarding quantitative and qualitative differences in levels of endocannabinoids, their receptors concentrations, and metabolizing enzymes in diverse tissue types for human patients of [8,9] as well as in mammalian models of neurodegenerative and related conditions [2,10,11,12]. Based on these spatial and temporal patterns of expression, endocannabinoids are thought to participate in diverse biological functions in specific cells layers of many tissues. The hypothesis is that the molecular targets of endocannabinoids have diverse locations to modulate physiological processes and behavior patterns in a cell- and tissue-specific manner.

Pre-clinical research on depression and neurodegenerative pathology compares the rodent frontal cortex after bulbectomy (removal of olfactory bulb) with pathological features seen in human brains of patients having neurodegenerative and/or neuropsychological disorders with aim of collecting comparable neuroanatomical, electrophysiological and molecular data [13]. For the last two decades, through studies exploring the advantages of various animal models for experimental manipulation, the olfactory system has emerged as a system to precisely analyze cellular, molecular, and neurological alterations correlated with specific patterns of behavior modulation [14,15,16].

Exposure to food odors by a social partner as a means of social transmission of food preferences evokes plasticity in olfactory bulb networks at the level of dendrodendritic synapse [17]. Specifically, such an experimental approach induces a glomerulus-specific long-term potentiation (LTP) at dendrodendritic synapses between GABAergic granule cells and mitral cells, the key output neurons in the olfactory bulb. The results indicate the existence of a synaptic substrate for a socially conditioned long-term memory at the first central relay for olfactory processing. Here, sensory cues are associated with social context through a socially relevant synaptic modification in the olfactory bulb. The LTP was blocked by deleting synaptotagmin, a calcium sensor or by deleting insulin-like growth factor 1 (IGF1) receptor in the olfactory bulb.

In a transgenic mouse model that specifically expresses a calcium sensor in olfactory bulb neurons, odor evoked activity shows a widespread lateral propagation due to blockage of dendrodendritic inhibition [18]. Despite the detection of cannabinoid type 1 receptor (CB1R) and cannabinoid type 2 receptor (CB2R) mRNA and protein in the olfactory epithelium, olfactory-mediated behavior remained normal in knockout mouse models of these receptors [19]. These findings suggest that the olfactory bulb is a site of synaptic plasticity with a functional role of the endocannabinoid system. The authors further show transport of such local effects to the limbic system.

Analysis of olfactory dysfunction in neurodegenerative pathology is becoming more important [20,21,22], especially since many neurodegenerative disorders exhibit olfactory deficits and olfactory dysfunction prior to the onset of neurodegenerative pathology [23]. Progressive tauopathy, i.e., the appearance of specific brain degeneration as a typical feature of Alzheimer’s disease, is detectable following olfactory dysfunction [24]. Olfactory deficits and dysfunction are known as prodromal symptoms of other neurodegenerative disorders including Parkinson’s disease (PD) as well as Alzheimer’s disease (AD) [25,26]. In addition, quantitative proteomic analysis of pathological alterations in the olfactory bulbs of patients suffering with progressive supranuclear palsy (PSP) and frontotemporal lobar degeneration, FTLD-TDP43, revealed an imbalance in survival signaling for both pathologies [27]. As olfactory bulb cell layers participate in initial information processing, they are the first contact point for external stimuli/stressors and serve as gateway for prion-like propagation of odorants, and pathogens as well as misfolded and unfolded proteins. Each of these may lead to neurodegenerative pathology, either individually or as combinatorial trigger [25]. Single cell transcriptome analysis during mouse olfactory neurogenesis in early development reveals that expression of olfactory receptor genes becomes progressively restricted to one gene per neuron in each mature neuron instead of several receptor genes that express in immature neurons [28,29]. Recently, the adult human olfactory bulb has become a popular and active site for the study of functional genomic influences on neurogenesis [30]. This is evident with CB1R and CB2R expression and functional studies using knockout mouse models [19]. In the mouse olfactory epithelium, immunohistochemistry shows diffuse CB1R protein expression, indicating protein localization in both neuronal and non-neuronal cell types. Whereas cell type specific CB2R protein expression remained ambiguous due to non-specific antibodies, it is evident simply by immunoblot, possibly due to CB2R expressing immune cells in the lamina propria [19]. These findings have established olfactory bulb cell layers as a dynamic site for modulation of molecular signaling at the single cell level throughout the lifetime of an organism.

While the functional participation of the endocannabinoid system in neuronal networks of the olfactory system is a newly emerging research focus, substantial research data is available about steps elaborating the functional dynamics of the endocannabinoid system in the limbic system. In contrast, the information and analysis regarding such steps in the olfactory system is very limited. In this review, we focus on the functional participation of the endocannabinoid system in neurodegenerative disorders. Specifically, we look at its impact on modulation of synaptic plasticity of neuronal networks in the olfactory bulb, where endocannabinoid system activation may influence and overlap with maintenance and alteration of synaptic plasticity that may lead to onset of neurodegenerative pathology. Our goal is to better understand how the signaling machinery in olfactory cell layers reacts to on-demand endocannabinoid system responses, to identify relevant pathways that contribute to neurodegenerative pathology, and to provide additional entry points to explore therapeutic intervention at the level of synaptic signaling.

## 2. Endocannabinoid Signaling System Components in Biological Functions

**Figure 1:**
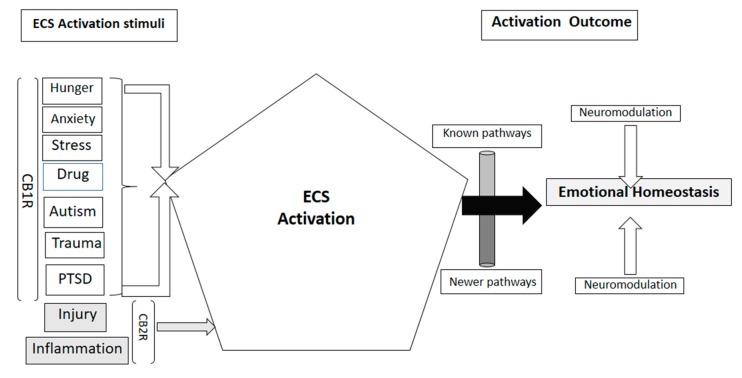
Schematic representation of stimuli that can activate the endocannabinoid system (ECS) and their impact through endocannabinoid receptors CB1R and CB2R, some pathways of neuromodulation are known, newer research studies are constantly revealing newer pathways.

Endocannabinoids are the main components of a lipid signaling network known as the endocannabinoid system. It responds to physiological alterations and environmental factors such as hunger, anxiety, stress, post-traumatic stress disorder (PTSD), and recreational drug use [1] while regulating energy balance [31]. Most important and extensively studied, the major endocannabinoids are N-arachidonoylethanolamine (anandamide, AEA) and 2-arachidonoylglycerol (2-AG) [32]. These compounds co-exist with quantitative differences based on tissue type and pathophysiological condition/s, which is an indication of their widespread biological role. Endocannabinoids are considered as bridging molecules that play a significant role in physiological and emotional homeostasis [33]. Several studies in human patients have demonstrated their fundamental role in the central nervous system [34] as well as in emotional homeostasis and cognition [9]. Primarily functioning through the activation of their own receptor systems CB1R and CB2R, they also interact with other receptors of diverse functions, such as transient receptor potential vanilloid 1 or TRPV1 channel [35], peroxisome proliferator-activated receptors (PPAR) α and γ [36] and orphan G protein-coupled receptor GPR55 [37]. CB1R, though widely expressed in many organs, has its highest expression in the brain [38], where it functionally modulates presynaptic neurotransmitter release and plays a significant role in memory function [16,39]. Earlier studies defined CB2R as peripheral receptor [40] due to its primary role in immunomodulation and anti-inflammatory effects of cannabis [41,42]. Recent studies have described it as a functional molecule in the brain and neurons impacted in Huntington’s disease (HD) [8], in AD, drug addiction [43,44], and in modulating stress induced depressive behavior using a mouse model [12,45]. These findings have strengthened the hypotheses regarding the pivotal role of the endocannabinoid system in neurodegenerative and psychoactive disorders. Figure 1 shows a schematic representation of various stimuli activating the endocannabinoid system for emotional and cognitive homeostasis.

## 3. Endocannabinoid Participation in Neurodegenerative Pathology

In addition to expression-based evidence of participation of CB1R and CB2R in AD, PD, and HD [8], mental disorders of anxiety and depression [9,46], psychosis [3] and PTSD [47], experimental evidence indicates that endocannabinoids participate in many other neuropathological disorders. A decrease in cannabinoid receptor mediated G-protein activation and binding activity in three brain regions (cortical, subcortical and cerebellar regions) of a schizophrenic rat model, with an especially high and significant reduction in a cerebellar membrane component, indicates an operational contribution of the endocannabinoid system in normal brain function [48]. Enhancing anandamide (AEA) signaling through inhibition of its degradation exerts prosocial effects in different animal models of autism spectrum disorders (ASD) [49]. Results from ex-vivo experiments provide evidence of wide alterations of CB1R and CB2R expression in several experimental models of multiple sclerosis (MS) and in patients affected by different clinical forms of MS [50,51,52,53,54,55]. Dysfunctional endocannabinoid signaling is also evident in a mouse model of Fragile-X syndrome [56]. An extensive review of available neuroimaging studies on the human brain indicates that the endocannabinoid system participates at multiple levels in the disruption of emotional processes, as well as executive and reward functions after drug use [57].

## 4. Endocannabinoid Mediated Signaling in the Olfactory Bulb

The first evidence of endocannabinoid dysregulation through the olfactory system comes from an experimental approach that uses bilateral bulbectomy in a rodent model. The model displays behavioral symptoms and neurochemical alterations that are clinically associated with schizophrenia and depression [58]. In the mouse olfactory epithelium, CB1R and CB2R mRNA and protein are detectable using RT-PCR and western blots. Immunohistochemistry and calcium imaging studies reveal that CB1R protein localizes in neurons and glial cells in the olfactory epithelium [19]. The authors observed a significant decrease of mature sensory olfactory neurons containing olfactory marker protein (OMP) in double knockout mouse model (CB1R^-/-^/CB2R^-/-^ mice), however they did not detect evidence of impaired olfactory function. Experimental evidence using an OMP knock out mouse model indicates OMP participation in segregation of axons in their target glomeruli and refinement of the olfactory bulb glomerular map [59]. In a lower vertebrate model *Xenopus laevis*, endocannabinoid mediated modulation is evident in the peripheral olfactory system. In hungry tadpoles, increased synthesis of endocannabinoid enhances the odor sensitivity of CB1R expressing olfactory neurons and enables them to locate food at lower odorant concentrations [60]. A similar association of CB1R signaling with hunger is evident in fasted mice, where a high level of CB1R is found in axon terminals of centrifugal cortical glutamatergic neurons that project to inhibitory granule cells of the main olfactory bulb [61]. CB1Rs affect olfactory bulb local circuits through cortical feedback as they function in a bidirectional mode where they modulate the ratio of inhibition and disinhibition in mitral cells of the main olfactory bulb [62]. Dietary restriction during pregnancy alters the endocannabinoid levels in the olfactory bulbs of pregnant female rats [63]. In humans, endocannabinoids participate in the reduction of olfactory function as they increase the olfactory threshold by activating G_i_ protein coupled receptors that reduce intracellular cAMP level and reduce olfactory acuity [64,65]. Tetrahydrocannabinol administration at 2mg/kg once every 48 h for 21 days in normal and olfactory bulbectomised rats reduced the hyperactivity in bulbectomised rats; administration of rimonabant, an anti-obesity drug and CB1R antagonist at slightly higher concentration of 5mg/kg had the same effect suggesting exogenous cannabinoids and modulating agents having significant impact as antidepressants [66]. Smaga et al. [46] confirmed similar observation in olfactory bulbectomised and genetically modified Wistar Kyoto (WKY) rats. These findings indicate the possibility of yet unidentified membrane receptors that might interact with CB1R and CB2R in vertebrate olfactory bulb cells to maintain normal neural plasticity and to decelerate the onset of degenerative neural pathology. Figure 1 depicts the known as well as the unknown and yet to be identified specific receptor pathways.

## 5. Olfactory Dysfunction and Neurodegenerative Pathology

Age-related decline in olfactory acuity and olfactory dysfunction in neurodegenerative disorders have long been reported [20,21,67,68]. In recent years, several studies have linked olfactory dysfunction with neurodegenerative pathology [69,70,71]. It has become an important clinical marker of neurodegenerative pathology along with other more direct clinical, biological and neuroimaging markers [72]. A direct correlation exists between quantitative smell tests and the amount of damage to forebrain neurotransmitter and neuromodulator circuits in patients suffering from a wide range of neurodegenerative diseases. It is a strong indicator of an impaired neural network connecting the olfactory bulb with the limbic system [73]. In general, correlation studies have used neuropathological markers, such as extracellular amyloid-β containing plaques, Lewy bodies and neurites made up of clusters of α-synuclein and intracellular neurofibrillary tangles of abnormally phosphorylated tau protein, to associate olfactory dysfunction with neurodegenerative diseases.

The first report of an association of olfactory dysfunction with AD came more than four decades ago [74] and has now emerged as predictor of AD associated dementia [75,76]. Additional research on other neurodegenerative conditions has linked olfactory dysfunction with PD [77,78,79], frontotemporal dementia (FTD) [80,81], other kinds of dementia [23], and mild cognitive impairment (MCI) [82]. For PSP, olfactory dysfunction has not been reported [83]. However, in PSP patients, the olfactory bulb specific stress-activated protein kinase (SAPK) pathway is turned off [27]. In contrast, in patients with the FTD variant FTLD (frontotemporal lobar degeneration dementia), another kinase pathway, the olfactory bulb specific phosphoinositide-dependent protein kinase 1 (PDK1) pathway shows disruption [27]. In both disorders, the authors show a common alteration in three other olfactory bulb signaling pathways: mitogen-activated protein kinases (MAPKs), calcium/calmodulin dependent protein kinase II (CAMK2II) and PKC (protein kinase C) pathway. Their quantitative proteome analysis detected a 1% difference in olfactory bulb proteome for normal controls and patients with FTDs. These observations are paving the way for a specific molecular analysis of signaling pathways in the olfactory bulb at the single cell level. Olfactory bulbectomy in rodents, an established procedure to model clinical depression and neurobiological features, provides clear evidence of resemblance between the perturbed frontal cortex in the bulbectomised rodents and the depressed human brain [13]. The characterization of cellular heterogeneity of the mouse olfactory bulb using single cell sequencing reveals differentially regulated and expressed genes as neuronal markers specific to adult born neurons. In mouse models of neurodegeneration, these may serve as molecular markers for different processes, such as synapse formation, synapse maintenance, and the neural plasticity of adult brain circuits [84].

## 6. Olfactory Capacity and Cellular Changes in Neurodegeneration

While olfactory dysfunction appears to be a prodromal symptom of multiple neurodegenerative disorders, it is also present to varying degrees in several neuropsychological disorders [34]. However, the cellular mechanisms leading to general degenerative neuropathology or disease specific pathogenesis have remained obscure. As functional MRI (fMRI) enables the simultaneous mapping of temporal and spatial patterns of neural activity in multiple brain regions, researchers have improved the technology to map the functional neuroanatomy of the olfactory system at multiple sites simultaneously in mouse models of neurodegeneration [85]. By using two different odorants and mapping the neural activity in the olfactory bulb as well as olfactory and limbic archicortex, the authors demonstrate odorant specific activation patterns in neurodegenerative mouse models. A closely related analysis using fMRI to evaluate olfactory dysfunction after olfactory stimulation along with standardized olfactory testing revealed a correlation in severity of olfactory deficits with the extent of neural networks recruited during olfactory stimulation as well as during pure sniffing [86]. These findings are showing the way to delineate cellular and molecular mechanisms of olfactory deficits in specific olfactory areas and to understand their impact on recruitment of occipital and cerebellar networks. Such an understanding is likely to provide the groundwork to analyze specific features of these network in order to predict disease prognosis as well as to apply and explore preventive and therapeutic interventions.

A characteristic neuropathological feature of AD is the appearance of neurofibrillary tangles consisting of hyperphosphorylated tau protein [87]. Studies in the human P301S tau transgenic mouse model reveal dependence of progressive tauopathy on functional deficits of the olfactory system. At around 3 months of age, mice show progressive tauopathy, neurodegeneration and impaired olfactory sensitivity to both social and non-social cues [24]. Recent experimental analysis using post-mortem olfactory bulb tissues from three control and three confirmed AD cases in their mid-seventies shows higher expression of P-tau in three olfactory bulb layers: mitral cell and granule cell layers as well as external plexiform layer (EPL) [26]. Additional experimental findings reveal that excessive P-tau expression in mitral cells of the P301S transgenic mouse model was associated with mitral cell loss at about nine months of age, while a decrease in mitral cell firing activity started at a young age of two months [26]. These findings place mitral cells as a putative target for drug therapy to ameliorate olfactory dysfunction. Experimental evidence has shown that while mitral cells lack cannabinoid receptors, they can be activated indirectly by stimulating CB1R present in GABAergic interneurons in the main olfactory bulb [6]. Possibly, specific kinase mediated pathways could alter the phosphorylation state of activator and repressor molecules, thereby contributing to activation of one or both cannabinoid receptors. CB1Rs localized in presynaptic terminals alter the release of GABA and glutamate neurotransmitters through voltage-gated ion channels as part of a retrograde messenger pathway [5].

Fragile-X syndrome (FXS) is an X-linked mental retardation disorder resulting from silencing of the FMR1 gene and loss of its protein product, FMRP, an mRNA binding protein that mainly acts as translational repressor. There is excessive basal protein synthesis in FXS individuals as well as in an FXS mouse model. Experimental approaches for rescuing FXS pathophysiology in Fmr-1 knock out mice reveal that genetic rescue normalizes excessive protein synthesis by regulating translation. FMRP appears to participate in resetting translational homeostasis in rescuing FXS pathophysiology [88]. A dysfunctional endocannabinoid system in FXS indicates an involvement of FMRP in the machinery activating endocannabinoid system receptors and other components [56]. This strengthens the hypothesis of the existence of multiple unknown components and unidentified pathways that interact with the endocannabinoid system and might contribute to emotional and cognitive homeostasis through the functional modulation of multiple neurotransmitters.

## 7. Conclusions and Outlook

In the preceding sections, we have summarized many studies that indicate an on-demand endocannabinoid system response in multiple tissues to a range of stimuli affecting widely different biological functions. While both neurodegenerative and neuropsychological disorders affect functional brain morphology and limbic system function in general, the olfactory system appears to have a pivotal role in the onset and progression of these conditions as olfactory deficits precede onset of visible pathology of neuronal degeneration as well as other symptoms. Specialized neuronal networks and unique expression patterns of olfactory receptors in the nasal epithelium and their connections to the olfactory bulb itself are among the first sites that are exposed to environmental altercations. Olfactory sensory neurons receive odor information and/or undesirable stimuli from the environment relaying those to the olfactory bulb. Each of these sensory neurons connects to specific glomeruli based on olfactory receptor expression. The prevailing hypothesis in the field of olfaction indicates the tight regulation of genes for olfactory receptors at multiple levels [28,29] as well as regulation through chromatin organization [89]

As adult neurogenesis continues in the olfactory bulb throughout the life span [90], it contributes to the cellular diversity of the olfactory bulb and influences the processing of odor information [91]. Odor information is processed in the main olfactory bulb by subtypes of inhibitory interneurons that arise as a result of adult neurogenesis in the subventricular zone of the lateral ventricles, integrate into available olfactory bulb circuits [92], and differentiate into granule cells and periglomerular cells [93]. Interneuron integration in the olfactory bulb is promoted by olfactory learning [94] but is decreased with olfactory deprivation [95]. These findings indicate that olfactory experience has a clear influence on existing olfactory bulb circuitry through integration of interneuron populations generated by adult neurogenesis. Using single cell RNA sequencing and computational modeling, diverse neuronal and non-neuronal cell types of the mouse olfactory bulb with distinct developmental programs have been identified. Experimental evidence further suggests that these molecular profiles, involving specific gene regulatory networks, reveal dynamic modulation in an activity dependent manner [84].

**Figure 2:**
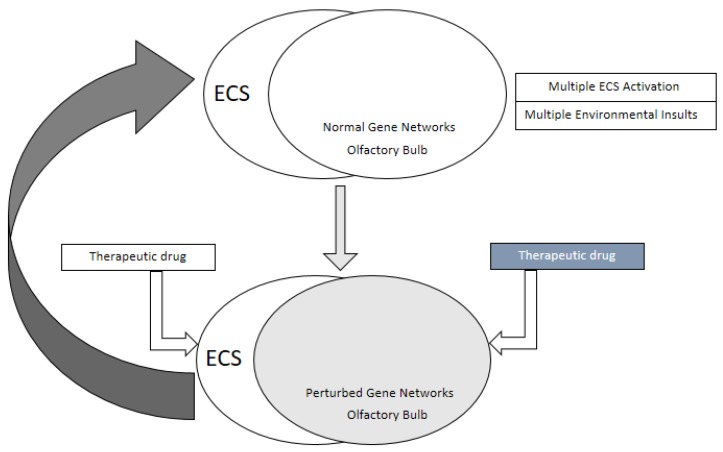
The endocannabinoid system (ECS) and the olfactory bulb as a site of therapeutic intervention for a functional olfactory pathway and neuronal homeostasis. ECS activation in response to diverse stimuli and environmental insults to the olfactory bulb affects overlapping physiological pathways and gene regulatory networks (top part of the figure) resulting in perturbed gene regulatory networks (shaded olfactory bulb in the lower half), a probable cause of onset and progression of olfactory deficits. The olfactory bulb and components of ECS can be used as therapeutic targets to restore homeostasis and physiological pathways as well as functional gene regulatory networks at a normal level. Just as there are multiple therapeutic targets, there could be at least two broader sets of therapeutic drugs: one set modulating ECS and the other set regulating olfactory bulb specific pathways at single cell level.

Based on such intricate molecular analysis, it is highly plausible that the existing olfactory circuitry is also affected by multiple factors such as aging, age-related inflammation, and associated physiological changes as well as other environmental insults that perturb the olfactory bulb gene regulatory networks as well as epigenome. It appears logical that recurrent on-demand activation of the endocannabinoid system could perturb gene regulatory networks in the olfactory bulb as well as the expression pattern of olfactory receptor neurons in the nasal epithelium leading to decreased olfactory acuity. This appears to precede the expression of neurodegenerative marker proteins and occurs before a full-fledged pathology can be detected in imaging studies [24,26]. Diverse neuronal and glial cell types in the olfactory bulb with their distinct transcriptome profile could be specific candidates as targets for drugs and genetic manipulation that may maintain normal gene regulatory networks and the epigenome as well as a provide greater stability at the level of chromatin, despite regulation by various physiological factors and on-demand activation of the endocannabinoid system. A schematic model of this hypothesis is represented in Figure 2. It provides intervention at two levels: (1) at the cellular level through direct genetic manipulation or drug-mediated modulation of regulatory pathways in diverse cell types of the olfactory bulb and (2) at the physiological level with drug induced alteration of the ECS activation, inhibition, and impact in various tissues in relation to the intensity of synaptic plasticity. Supporting evidence for such a model comes from studies of the drug fluoxetine. In a mouse model of depression, fluoxetine reverses olfactory bulbectomy induced depressive behavior by increasing neurotransmitter acetylcholine activity in the limbic system [96] and alters CB1R expression pattern in the prefrontal cortex with chronic drug use [97]. Additional studies including transcriptome profiling of cell types in the olfactory bulb of various neurodegenerative and neuropsychiatric conditions in mouse models as well as in human patients will enable further detailed analysis of gene regulatory networks that are specific for the pathology of different disorders as well as for recreational, therapeutic, or addictive drug usage.
